# The influence of sport motivation on college students’ subjective exercise experience: a mediation model with moderation

**DOI:** 10.3389/fpsyg.2023.1219484

**Published:** 2023-08-31

**Authors:** Fengbo Liu, Ning Li

**Affiliations:** ^1^School of Physical Education, Zhengzhou University of Light Industry, Zhengzhou, Henan, China; ^2^School of Physical Education, Putian University, Putian, Fujian, China

**Keywords:** sport motivation, subjective exercise experience, sport-confidence, feelings of inadequacy, mediation model with moderation

## Abstract

**Background:**

The degree to which an individual experiences a positive emotional state after exercise is a measurement of how much enjoyment sports bring to the individual. This can also be seen as the individual’s essential motivation for engaging in sports, and an indirect means of improving the individual’s physical health. Therefore, it is necessary to explore the factors that affect college students’ subjective exercise experience and their effecting mechanism, thereby providing a basis for promoting college students’ positive emotional experience after exercise.

**Methods:**

A questionnaire survey on 600 college students was conducted to examine the mediating effect of sport-confidence on the relationship between college students’ sport motivation and their subjective exercise experience, and to investigate whether this process is moderated by the feelings of inadequacy.

**Results:**

The indirect effect of sport-confidence was significant (95% CI [0.133, 0.276]), and the index of moderated mediation Bootstrap 95% CI [0.003, 0.017] did not contain 0.

**Conclusion:**

The results indicated that: (1) sport-confidence had a partial mediating effect between college students’ sport motivation and their subjective exercise experience; (2) the mediating effect of sport-confidence was moderated by the feelings of inadequacy, and the feelings of inadequacy moderated the latter half path of the mediating process of sport motivation - sport-confidence - subjective exercise experience. Therefore, the influence of college students’ sport motivation on their subjective exercise experience is a moderated mediating model.

## 1. Introduction

College students are senior professionals trained by the country, and they are the mainstay of driving social progress, so their physical and mental health is being taken more and more seriously by all sectors of society. With the continuous development of contemporary medicine and positive psychology, people’s focus of attention has gradually changed from individual’s behavior to individual’s mental health. Mental health not only refers to having no mental illness, but also to having a positive mental health state (Eaton and Ryan, 2017). “Subjective exercise experience” refers to the degree to which an exerciser subjectively experiences positive emotional states, negative emotional states, and physiological consumption after exercise (Sun et al., 2021). The degree to which an individual experiences a positive emotional state after exercise is a measurement of how much enjoyment sports bring to the individual. This can also be seen as the individual’s essential motivation for engaging in sports (Nägel and Sonnentag, 2013), and an indirect means of improving the individual’s physical health. Therefore, it is necessary to explore the factors that affect college students’ subjective exercise experience and their effecting mechanism, thereby providing a basis for promoting college students’ positive emotional experience after exercise.

Motivation is the direction and reinforcement of all biological individuals for their hopes (Bauer et al., 2016). “Sport motivation” refers to the internal dynamics that motivates people to participate in sports activities, driven by the needs of exercise (Tušak et al., 2022). Numerous studies on subjective exercise experiences have revealed that the individuals with a stronger sport motivation experience a higher level of positive emotion during exercise (Skinner and Brewer, 2004).

However, it is far from enough to only discuss the direct relationships among variables. It is necessary to further explore the internal mechanism of sport motivation affecting subjective exercise experience on the basis of previous studies. The discussion on the mediating effect is conducive to clarifying how sport motivation affects subjective exercise experience, while the study on the moderating effect is conducive to clarifying when such influence takes effect. Furthermore, the study on the mediation with moderation not only can reveal whether the mediating process is moderated by the moderating variables, but also can answer “how” the independent variable affects the dependent variable and “when” the influence is stronger or weaker (Edwards and Lambert, 2007).

On the basis of literature review, this study believes that sport-confidence may be an important mediating variable in the influence of sport motivation on subjective exercise experience. Confidence is the expression of a person’s self-worth. “Sport-confidence” refers to the level of belief and confidence that an individual feels or actually shows on the spot and believes that he/she can fully utilize his/her abilities in sports (Machida et al., 2017), which affects the way an individual responds to the current sport environment. Young people who have experienced early success become more confident, feel more self-worth, and have a stronger motivation to pursue significant accomplishments, so an individual’s sport-confidence is significantly positively correlated with his/her sport motivation (Levy et al., 2015). The studies on cognitive behaviors of individuals with different confidence levels have revealed that the individuals with high confidence tend to actively process information, while the individuals with low confidence are more associated with negative emotions and behaviors. In the multi-dimensional anxiety model proposed by Martens et al. sport-confidence is seen as a lack of negative competitive emotion (Zurlo et al., 2020). Besides, some scholars propose that sport-confidence should be regarded as a concept relatively independent of negative competition emotion, and believe that there is a clear distinction between negative emotion cognition and sport-confidence (Chun et al., 2023). To sum up, the individuals with a higher sport motivation have a relatively higher sport-confidence, which further deepens the level of positive emotional experience after exercise. Therefore, this study proposes hypothesis 1 that sport-confidence is a mediating variable between sport motivation and subjective exercise experience.

Although sport motivation may have an important influence on subjective exercise experience through sport-confidence, there may be some differences in this kind of influence, so it is necessary to examine whether this mediating effect is affected by other variables. Considering that the sport-confidences of college students with the same level of sport motivation are not exactly the same, and that the subjective exercise experiences of college students with the same level of sport-confidence are not exactly the same, it is necessary to investigate whether other variables moderate the relationship between sport-confidence and subjective exercise experience.

College students are a special group whose mental health is far from satisfactory. They are a group with a high incidence of psychological disorder (Masuda et al., 2014). Psychological disorders affect their positive emotional experience after exercise. Therefore, we can think about whether the influence of sport-confidence on subjective exercise experience varies depending on individuals’ feelings of inadequacy. On the one hand, when the individuals have weak feelings of inadequacy, the individuals with a higher sport-confidence are more likely to experience positive emotions after exercise than the individuals with a lower sport-confidence (Koehn et al., 2013). On the other hand, from the definitions of sport-confidence and feelings of inadequacy, it is believed that positive factors usually lose their influence in an environment with strong feelings of inadequacy. In other words, when the individuals have weak feelings of inadequacy, the individuals with positive qualities experience good; conversely, when the individuals have strong feelings of inadequacy, their experience of positive emotions would decline rapidly. Many empirical studies provide the basis for this statement. For example, when the individuals have strong feelings of inadequacy, their level of belief and conviction in believing that they can fully utilize their abilities in sports is low (Hu et al., 2019). Under such a circumstance, regardless of the level of college students’ sport-confidence, their experience of positive emotions after exercise would be low. In other words, the feelings of inadequacy moderate the relationship between sport-confidence and subjective exercise experience. Therefore, this study proposes hypothesis 2 that the feelings of inadequacy moderate the mediating effect of sport-confidence.

So far, few studies have tested whether the feelings of inadequacy have a moderating effect on the relationship between sport motivation and subjective exercise experience (including direct or indirect relationship), and what is the specific mode of the moderating effect. Considering that there is still a lack of research evidence up to now, this study only makes exploratory analysis on the moderating effect of the feelings of inadequacy, but does not make a clear judgment on its specific model.

In summary, this paper proposes a mediation model with moderation for the purpose of promoting college students’ positive emotional experience after exercise. This study mainly covers two aspects: (1) exploring whether sport-confidence has a mediating effect on the relationship between sport motivation and subjective exercise experience; (2) examining whether the feelings of inadequacy have a moderating effect on the mediating effect, that is, whether the mediating process of sport motivation on subjective exercise experience through sport-confidence is moderated by the feelings of inadequacy. This study focuses on whether the feelings of inadequacy moderate the latter half path of this process.

## 2. Methods

### 2.1. Sample

The samples for this study were selected from college students of Northeast University, Zhengzhou University, Shaanxi Normal University, Tianjin Foreign Studies University, Henan University of Technology, Zhengzhou University of Light Industry and other universities. The criteria for selecting the samples: (1) at least 18 years old; (2) having no medical history of mental disorders; and (3) non-sports or art major. All of them were told that they would participate in a study on psychological survey. A total of 600 questionnaires were distributed, and 560 valid questionnaires were recovered after eliminating invalid ones. The recovery rate of valid questionnaires was 93.3%.

### 2.2. Instruments

#### 2.2.1. Sport motivation inventory

This study employs the sport motivation inventory (SMI) prepared by Zhang (2000). The SMI is divided into three dimensions: participation tendency, avoidance tendency and sport motivation. In this research, the result of reliability test shows that the Cronbach’s α of the Sport Motivation Inventory (SMI) with six items is 0.69, which basically meets the requirement for internal consistency.

#### 2.2.2. State sport-confidence inventory

This study employs the state sport-confidence inventory (SSCI) prepared by Vealy (1986). The SSCI includes 13 items, and it uses the nine-point scale, representing the process of change from low confidence (1 point) to high confidence (9 points). After summarizing the scores of 13 items, the higher the total score is, the stronger the sport-confidence is. In this research, the result of reliability test shows that the Cronbach’s α of the SSCI is 0.96.

#### 2.2.3. The feelings of inadequacy scale

This study employs the physical strength inadequacy subscale of the feelings of inadequacy scale (FIS) prepared by Church et al. (1980). The Subscale includes 5 items, and uses the five-point scale, in which a low score represents strong feelings of inadequacy. In this research, the result of reliability test shows that the Cronbach’s α of the subscale is 0.87.

#### 2.2.4. The subjective exercise experience scale

This study employs the positive wellbeing subscale of the subjective exercise experience scale (SEES) prepared by Mcauley and Courneya (1994). The Subscale includes 5 items, and uses the seven-point scale, in which the higher score is, the stronger positive wellbeing experience is. In this research, the result of reliability test shows that the Cronbach’s α of the subscale is 0.83.

### 2.3. Procedures

Through online recruitment, 229 male students and 331 female students (including 188 freshmen, 124 sophomores, 65 juniors, 95 seniors, and 88 graduate students) were invited and abroad from June 1 to June 15, 2022. To explore the effect of various demographic characteristics on the association between the sport motivation and subjective exercise experience, several choice (e.g., What is your gender?) and fill-in (e.g., How old are you?) questions were designed for all participants. The study was approved by the Ethics Committee of Zhengzhou University of Light Industry, and all participants completed an informed consent form before filling out the questionnaire.

### 2.4. Statistical methods

The basic data questions and the above-mentioned four scales are bound in a volume to form the questionnaire, and the SPSS 19.0 statistical software is employed to process and analyze the data. Bivariate correlation was used to test the relationship between gender, age, sport motivation, sport-confidence, feelings of physical strength inadequacy, and positive wellbeing experience. The macro program PROCESS of SPSS was used to test the mediating effect of sport-confidence and the moderating effect of the feelings of physical strength inadequacy. The number of Bootstrap samples was 5,000. Under the 95% confidence interval, gender and age were all controlled as covariates.

## 3. Results

### 3.1. The description and the correlation of variables

The descriptive statistics and related matrices for the variables in this study are as shown in Table 1. The sport motivation (*r* = 0.50, *p* < 0.01), sport-confidence (*r* = 0.47, *p* < 0.01), and feelings of physical strength inadequacy (*r* = 0.17, *p* < 0.01) are significantly positively correlated with positive wellbeing experience.

**Table 1:** The description and the correlation of variables (*N* = 560).

Variables	*M*	*SD*	1	2	3	4	5	6
1. Gender	1.59	0.49	–					
2. Age	2.24	0.48	0.02	–				
3. Sport motivation	15.48	3.09	−0.11**	0.08	–			
4. Sport-confidence	71.24	20.07	−0.17**	0.01	0.35**	–		
5. Feelings of physical strength inadequacy	27.09	3.94	−0.05	0.00	0.12**	0.15**	–	
6. Positive wellbeing	19.61	4.86	−0.06	0.09*	0.50**	0.47**	0.17**	–

For gender, male = 1, female = 2; for age, age under 18 = 1, age 18 ~ 22 = 2, age 23 ~ 26 = 3, and age over 26 = 4. *p < 0.05, **p < 0.01, ***p < 0.001, the same hereinafter.

### 3.2. The influencing mechanism on subjective exercise experience

**Figure 1:**
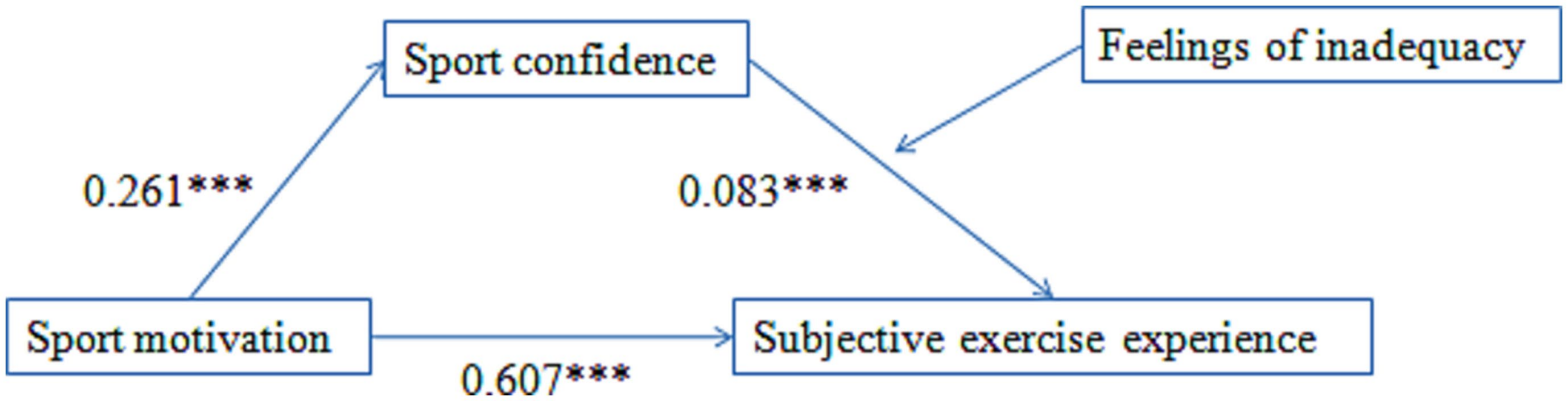
The mediation model with moderation.

From Table 2, it can be found that the indirect effect of sport-confidence was significant (95% CI [0.133, 0.276]); after the mediating variable entered the equation, the direct effect Bootstrap 95% CI [0.495, 0.720] did not contain 0, which indicated that sport-confidence had a partial mediating effect between sport motivation and subjective exercise experience. Therefore, hypothesis 1 is supported. Besides, the index of moderated mediation Bootstrap 95% CI [0.003, 0.017] did not contain 0, which indicated that the feelings of physical strength inadequacy have a moderating effect on the relationship between sport-confidence and subjective exercise experience. Therefore, hypothesis 2 is supported (Figure 1).

**Table 2:** Summaries of process effect test.

Dependent variable: positive wellbeing experience	Effect/index	SE	95% CI LL	95% CI UL
Gender	0.42	0.34	−0.253	1.100
Age	0.56	0.35	−0.129	1.244
Sport confidence	0.01	0.03	−0.045	0.061
Feelings of inadequacy	−0.40	0.10	−0.592	−0.209
Direct effect of sport motivation	0.61	0.06	0.495	0.720
Indirect effect of sport-confident	0.20	0.04	0.133	0.276
Index of moderated mediation	0.01	<0.01	0.003	0.017

In order to further analyze the trend of the moderating effect of the feelings of physical strength inadequacy, the feelings of physical strength inadequacy were grouped based on the mean and plus/minus one SD from mean (Table 3).

**Table 3:** Conditional indirect effect of sport-confidence.

Mediator	Feelings of inadequacy		Effect	Boot SE	95% CI LL	95% CI UL
Sport-confidence	M − 1SD	14.80	0.15	0.03	0.087	0.224
	M	20.20	0.20	0.04	0.133	0.276
	M + 1SD	25.60	0.25	0.05	0.164	0.348

Table 3 showed “how” the influence of sport-confidence on positive wellbeing experience is moderated by the feelings of physical strength inadequacy. The effect measures the influence of sport-confidence on positive wellbeing experience. The effect shows that when the feelings of physical strength inadequacy are weak, the effect of sport-confidence on positive wellbeing experience is significant (effect = 0.15, 95% CI [0.133, 0.276]). When the feelings of physical strength inadequacy are strong, the effect of sport-confidence on positive wellbeing experience is still significant (effect = 0.25, 95% CI [0.164, 0.348]).

## 4. Discussion

Recently, the psychological problems of college students have become major concerns worldwide (Xu, 2022). From a subjective view, the subjective exercise experience as the individual’s essential motivation for engaging in sports satisfied college students’ psychological health needs partly. Motivation is an internal driving force that makes people move towards the desired goal, and it is an important psychological state for individual development. The current study found that sport motivation promotes positive emotions in subjective exercise experience, that is, the individuals with a stronger sport motivation experience a deeper level of positive emotions after exercise, which is consistent with previous conclusions (Davis et al., 2021), thereby increasing the generalizability of the relationship between sport motivation and subjective exercise experience (Kim et al., 2010). On the basis of verifying direct relationships, this study constructs and tests a mediation model with moderation, and explores the mechanism of the influence of sport motivation on subjective exercise experience.

### 4.1. The direct effect of sport motivation

This study has found that sport motivation can independently and significantly positively predict positive subjective exercise experience. In other words, the higher the level of college students’ sport motivation is, the higher their level of positive subjective exercise experience is, which is consistent with the findings from the studies of Xiao et al. (2008).

As an internal psychological motivator that keeps students engaged in physical education learning and physical exercise activities, sport motivation presents a new thinking for explaining how psychological factors affect college students’ individual behaviors. Sport motivation focuses on the more advanced structures behind several important psychological characteristics. This helps to better understand the relationships between psychological factors and outcome variables and achieve more effective predictions of them. In this study, sport motivation includes three basic components: participation tendency, avoidance tendency, and total score for motivation. With these three psychological factors, college students can respond positively to external stimuli during sports, effectively control and regulate their own emotions, and fully engage in sports. These positive psychological and behavioral tendencies can significantly reduce negative reactions such as psychological distress and fatigue among college students (Zeng and Liu, 2013), which would undoubtedly have a significant effect on improving positive wellbeing. This indicates that improving college students’ sport motivation through interventions may be an effective way to enhance their positive wellbeing experience after exercise.

### 4.2. The mediating effect of state sport-confidence

This study introduces sport-confidence to explore the specific mechanism by which sport motivation affects subjective exercise experience. The results reveal that sport motivation is positively associated with sport-confidence. Currently, many researchers have same perspective regarding the relationship between sport motivation and sport-confidence, that is, sport-confidence growth results in an increase in sport motivation (Brennan et al., 2021). Sport-confidence can be considered to be an individual’s belief toward their athletic capabilities. Bandura’s conceptual model of self-efficacy brings together concepts of confidence and motivation (Spittle et al., 2022). That is, individuals with strong sport confidence are more willing to participate in sports.

In addition, sport-confidence has a significant positive effect on college students’ positive wellbeing experience after exercise, which is consistent with previous research findings (Standage et al., 2008). Current studies show that individuals with high levels of sport-confidence can actively overcome negative emotions during exercise. Individuals with high levels of sport-confidence firmly believe that exercise can bring mental health benefits to themselves, and can obtain pleasant emotional experiences and maintain good peer relationships through exercise (Scher and Osterman, 2002).

More importantly, this study has found that sport-confidence is a “black box” between sport motivation and subjective exercise experience. In the process where sport motivation promotes positive emotional experiences after exercise, sport-confidence has a partial mediating effect, that is, the influence of sport motivation on college students’ subjective exercise experience is achieved on the one hand through a direct approach, and on the other hand, it is achieved through the indirect approach of influencing sport-confidence. Here, sport-confidence acts as a “bridge,” reflecting not only its relationship with sport motivation, but also its relationship with subjective exercise experience. It has answered “how” or “why” sport motivation would affect subjective exercise experience. Therefore, sport-confidence is an important intrinsic factor in the influence of sport motivation on college students’ subjective exercise experience.

First of all, motivation improves the confidence level of college students, which is the same as previous conclusions (Spittle et al., 2022). The expansion of cognition and behavior caused by sport motivation helps college students increase their engagement and persistence in sports, and constantly strengthens the level of individuals’ sport-confidence (Liu et al., 2011). Moreover, the college students with a higher level of sport motivation have more intense and frequent sports experiences, and experience more fun and the joy of success during sports. Through self-evaluation, these successes and experiences make them aware of their own value and develop a sense of competence, thereby enhancing their level of sport-confidence (Cheng et al., 2013). Secondly, sport-confidence suppresses college students’ experience of negative emotions after exercise, which is consistent with previous research conclusions (Yue et al., 2014). In other words, sport-confidence can promote college students’ experience of positive emotions after exercise. To sum up, sport motivation not only directly affects college students’ subjective exercise experience, but also indirectly affects their subjective exercise experience by influencing their sport-confidence.

Therefore, the results support our initial prediction that the relationship between sport motivation and the subjective exercise experience is mediated by sport-confidence. The discovery of this mediating effect is of practical significance. On the one hand, it reminds us that the influence of sport motivation on subjective exercise experience is complex. Sport motivation not only directly affects subjective exercise experience, but also indirectly affects subjective exercise experience through other channels. On the other hand, the discovery of this mediating effect would help promote college students’ experience of positive emotions after exercise.

### 4.3. The moderating effect of the feelings of inadequacy

This study has examined whether the mediating effect of sport-confidence varies depending on college students’ different feelings of physical strength inadequacy. The mediation model where sport motivation affects subjective exercise experience through sport-confidence is further deepened and developed by adding the qualifications “when” is stronger or weaker. The results show that the feelings of inadequacy have a significant negative influence on college students’ experience of positive emotions after exercise, which is consistent with previous research conclusions (Chen and Danish, 2010).

What is especially important is that this study has found that the feelings of inadequacy moderate the influence of sport-confidence on subjective exercise experience, that is, the influence of sport-confidence on positive wellbeing experience would increase as the feelings of inadequacy increases. When they have stronger feelings of inadequacy, college students are more likely to feel alone and have more avoidance and distress for social interaction (Loukas et al., 2010), which cause them to reduce their engagement and persistence in sports, and then they gradually lack state sport-confidence, thus reducing their experiences of positive emotions after exercise. The data in this study also supports for such arguments. In other words, as a factor, the feelings of inadequacy weaken the promoting effect of sport-confidence on positive emotional experience after exercise. From another perspective, as the feelings of physical strength inadequacy increase, the positive wellbeing experience of the college students with a higher level of sport-confidence would decrease. Therefore, reducing the feelings of physical strength inadequacy can benefit the college students with a higher level of sport-confidence. In addition, as college students’ feelings of physical strength inadequacy weaken, sport-confidence would significantly improve the degree of college students’ positive wellbeing experience.

The implications of this model for us are: First, we should focus on cultivating college students’ state sport-confidence trait; second, we should not exaggerate the role of sport-confidence trait. When they have stronger feelings of inadequacy, the effect of sport-confidence on improving college students’ positive wellbeing experience is relatively limited, so attention should also be paid to protecting college students’ self-esteem. Therefore, for the purpose of enhancing college students’ positive wellbeing experience after exercise, on the one hand, it is necessary to strengthen individuals’ sport-confidence; on the other hand, it is necessary to attach importance to protecting college students’ self-esteem.

In summary, the mediation model with moderation proposed in this study has revealed in greater depth the effecting mechanism between sport motivation and college students’ subjective exercise experience. Sport motivation affects college students’ subjective exercise experience through sport-confidence. Such effect decreases as the feelings of inadequacy increases. The findings from this study have two theoretical implications. First, the mediation model of “motivation - confidence - positive experience” is supported in a new research context, which is a constructive repetition of previous studies. Second, the above-mentioned mediation model is further deepened and developed by adding the qualifications “when” is stronger or weaker, which effectively integrates sport motivation and feelings of inadequacy into a theoretical frame work. In fact, this “integrated” research approach has garnered more and more attention in recent years (Podsakoff et al., 2003), because it not only enriches the concept of “influencing factor,” but also improves the explanatory power of the model. This is also the value of the mediation model with moderation, which is different from the simple mediation or moderation model. This study has revealed the intrinsic mechanism by which sport motivation affects college students’ subjective exercise experience. Moreover, it has certain reference value for the practical work of improving the campus atmosphere and improving students’ physical health. First, this study suggests that school physical education workers must attach importance to the role of sport motivation in enhancing college students’ positive wellbeing experience, and promote college students’ positive wellbeing experience after exercise by taking interventions and enhancing sport motivation. Second, sport-confidence has mediating effects between sport motivation and positive wellbeing experience. This suggests that we can enhance college students’ positive wellbeing experience by improving their sport-confidence. Moreover, there is no conflict between students’ social–emotional needs and the need to enhance their physical fitness. Finally, this study has found that the indirect effect of sport motivation on college students’ positive wellbeing experience through sport-confidence is attenuated by strong feelings of physical strength inadequacy. Therefore, in practical work, it is necessary to distinguish the level of college students’ feelings of inadequacy, to put more limited energy on the individuals with strong feelings of inadequacy, and to help them enhance their sport-confidence and improve their positive wellbeing experience.

### 4.4. Research limitations and prospects

There are still some limitations in this study. First, on the basis of in-depth investigation of existing studies, this study starts from selecting two specific concepts including sport motivation and subjective exercise experience, and explores the relationship between participation tendency, total score for sport motivation and positive wellbeing experience. However, as mentioned above, sport motivation also includes avoidance tendency; and subjective exercise experience also includes psychological distress and fatigue. In order to further expand the relationship between sport motivation and subjective exercise experience, we suggest that future research can focus on the relationship between sport motivation and the other three specific dimensions of subjective exercise experience, or incorporate participation tendency, total score for sport motivation, and positive wellbeing experience into the scope of discussion, and compare the strengths or weaknesses of different relationships between sport motivations and college students’ subjective exercise experiences.

Second, from a methodological point of view, this study mainly uses the questionnaire method for research, which may not provide sufficient evidence for causal relationships among variables. In order to solve this problem, we collected data from multiple locations at multiple time points to test our theoretical models. This can reflect causal relationships to a certain extent and effectively address the effects of common method biases (Podsakoff et al., 2003). However, we suggest that future research should use an experimental approach to explore the influence of sport motivation on subjective exercise experiences, thereby improving the internal validity of the results. For example, researchers can manipulate the level of sport motivation (such as giving some benefits to the test subjects who participate in the exercise), and then test the effects on the test subjects’ state sport-confidence and subjective exercise experience, thus establishing the causal relationships.

## 5. Conclusion

In conclusion, our research found that: (1) Sport-confidence has a partial mediating effect between college students’ sport motivation and their subjective exercise experience; (2) the mediating effect of sport-confidence is moderated by the feelings of inadequacy, and the feelings of inadequacy moderate the latter half path of the mediating process of sport motivation - sport-confidence - subjective exercise experience. The influence of sport-confidence on subjective exercise experience decreases as the feelings of inadequacy increase.

Compared to previous studies, this study explored the relationship between sport motivation, subjective exercise experience, sport confidence, and feelings of inadequacy, and verifying the moderating role of feelings of inadequacy in the mediating process. This study clarified the influencing factors and mechanisms of college students’ subjective exercise experience, and providing a theoretical basis for improving their subjective exercise experience in the future, which may provide guidance for sport psychologists.

## Data availability statement

The original contributions presented in the study are included in the article/supplementary material, further inquiries can be directed to the corresponding author.

## Ethics statement

The studies involving humans were approved by Ethics Committee of Zhengzhou University of Light Industry. The studies were conducted in accordance with the local legislation and institutional requirements. The participants provided their written informed consent to participate in this study.

## Author contributions

FL: conceptualization, writing – original draft, and writing – review and editing. FL and NL: data curation and investigation. All authors contributed to the article and approved the submitted version.

## Funding

This study was supported by Doctor Research Fund from Zhengzhou University of Light Industry (2022BSJJSK12), Social Science Research Project in Zhengzhou (ZSLX20231702), Research of Educational Science Planning in Henan Province (2023YB0105), Research on Humanities and Social Sciences in Universities in Henan Province (2024-ZZJH-255), and Doctor Research Fund from Putian University (2023051). The funders provided support for the preparation and publication of this paper.

## Conflict of interest

The authors declare that the research was conducted in the absence of any commercial or financial relationships that could be construed as a potential conflict of interest.

## Publisher’s note

All claims expressed in this article are solely those of the authors and do not necessarily represent those of their affiliated organizations, or those of the publisher, the editors and the reviewers. Any product that may be evaluated in this article, or claim that may be made by its manufacturer, is not guaranteed or endorsed by the publisher.
